# WHAT IS MEMORY CARE IN ASSISTED LIVING?

**DOI:** 10.1093/geroni/igae098.1659

**Published:** 2024-12-31

**Authors:** Lindsey Smith, Eric Jutkowitz, Kali Thomas

**Affiliations:** Oregon Health & Science University, Portland, Oregon, United States; Brown University, Brown University, Rhode Island, United States; Johns Hopkins University, Baltimore, Maryland, United States

## Abstract

Memory care is the most common type of specialized care provided in assisted living (AL) settings. This specialized care caters to people living with dementia and other forms of cognitive impairment. However, what constitutes specialized memory care varies according to who and how the setting is defined. We use the lenses of the state, industry, and consumers to describe the prevalence of ALs with memory care across the US. We created a national directory of ALs and their state licenses to identify those ALs designated as providing specialized memory care by the licensing agency, classified according to whether the state holds these ALs to additional standards or simply requires disclosure. To identify AL communities considered memory care by industry professionals, we relied on a dataset of AL organizations from the National Investment Center (NIC), a nonprofit organization focused on providing data, analytics, and connections to the senior housing and care industries. Finally, to view AL communities through the lens of (potential) residents and their families, we used business advertisement descriptions from Data Axle, a business advertising database. We found that 28% of the 13,715 large (greater than 25 beds) AL communities in our national AL directory were identified as memory care by state licensing agencies, 60% by NIC, and 26% by Data Axle. Our findings raise questions regarding how the care provided in these settings may vary based on who defines memory care.

